# Genetic Diversity of White-spotted Rabbitfish (*Siganus canaliculatus*) on Different Seagrass Habitats in Inner Ambon Bay, Indonesia Based on Mitochondrial CO1 Sequences

**DOI:** 10.21315/tlsr2024.35.1.15

**Published:** 2024-03-30

**Authors:** Husain Latuconsina, Nurlisa A. Butet, Ridwan Affandi, M. Mukhlis Kamal, Syamsul Bachry, Agus Alim Hakim

**Affiliations:** 1 Department of Biology, Faculty of Mathematics and Natural Sciences, Universitas Islam Malang, Dinoyo, Malang 65144, Indonesia; 2 Department of Aquatic Resources Management, Faculty of Fisheries and Marine Science, IPB University, FPIK Bld. 3rd Floor, Kampus IPB Dramaga, Bogor 16680, Indonesia; 3 Program Study of Biology, Universitas Pahlawan Tuanku Tambusai, No. 23 Bangkinang Kota, Kampar, Riau 28412, Indonesia

**Keywords:** Genetic Differentiation, Phylogenetic, Genetic Distance, Genetic Diversity, Single Nucleotide Polymorphisms, SNPs

## Abstract

This study aims to analyse the genetic diversity of *Siganus canaliculatus* in the Inner Ambon Bay (IAB) waters. DNA of *S. canaliculatus* specimens collected from IAB was extracted from tissues using a Tissue Genomic DNA Mini Kit, and partial CO1 genes were amplified using pair of universal primers. Genetic distances were determined by Kimura 2-parameter, and phylogenetic trees were constructed using the neighbour-joining method in MEGA 10.2.2 software. Arlequin software was used to analyse Fixation Index (Fst) and Analysis of Molecular Variance (AMOVA). There are three SNPs of *S. canaliculatus* from IAB that distinguish GenBank sequence data from *S. canaliculatu*s. In Tanjung Tiram population group, contained three specific 677 (A), 679 (G), 703 (T) sites and two 693 (G), 714 (A) sites for the Nania population. Haplotype and nucleotide diversity of each population range from 0.000 to 1,000 and 0.000 to 0.004. Intra- and inter-population genetic differentiation were 21.19% dan 78.81%, respectively. Intra- and inter-population genetic distances were in range of 0.40–1.13 and 0.00–0.37, respectively. The pattern and direction of tidal currents as a link or barrier to spatial distribution and connectivity of *S. canaliculatus* larvae between seagrass habitats, as well as the presence of different anthropogenic pressures in each seagrass habitat, are thought to influence the genetic characteristics (genetic diversity, genetic variation, genetic differentiation and genetic distance) of *S. canaliculatus* populations in IAB waters. The results of this study provide information about the urgency of habitat-based fisheries management to support sustainable utiliation.s

**Highlights**

- The genetic diversity (haplotype and nucleotide variability) of white-spotted rabbitfish *(Siganus canaliculatus)* population living in different seagrass habitats in Inner Ambon Bay (IAB) waters.
- Intra- and inter-population genetic differentiation of *Siganus canaliculatus* were 21.19% dan 78.81%, respectively. Intra- and inter-population genetic distances were in range of 0.40–1.13 and 0.00–0.37.
- The pattern and direction of tidal currents as a link or barrier to spatial distribution and connectivity of *S. canaliculatus* larvae between seagrass habitats, as well as the presence of different anthropogenic pressures in each seagrass habitat, are thought to influence the genetic characteristics (genetic diversity, genetic variation, genetic differentiation, and genetic distance) of *S. canaliculatus* populations in IAB waters.

## INTRODUCTION

The white-spotted rabbitfish (*Siganus canaliculatus* Park, 1797) is a teleosts fish belonging to the family Siganidae and order Perciformes (Woodland 2001). This rabbitfish is widely distributed in the Indo-Pacific region by utilising tidal rhythms in three important habitats, namely mangroves, seagrass beds and coral reefs (Allen & Erdmann 2012; Latuconsina *et al*. 2013; Latuconsina *et al*. 2023). In the Inner Ambon Bay (IAB hereafter), *S. canaliculatus* occurs in a variety of seagrass habitats with different densities and diversity of vegetation (Ambo-Rappe *et al*. 2013; Latuconsina *et al*. 2020a), enough to influence and determine the variation of growth and reproduction biology (Latuconsina *et al*. 2022).

The IAB waters are naturally separated from Outer Ambon Bay (OAB) by a narrow inlet (sill) with a depth of ±12 m, causing the water mass circulation not to run smoothly (Basit *et al*. 2012; Noya *et al*. 2016a; Saputra & Lekalette 2016; Salamena *et al*. 2021). Various anthropogenic activities threaten the existence of seagrass habitats in IAB, such as garbage disposal, organic waste and dredging of beach sand (Evans *et al*. 1995; Selano *et al*. 2009; Manullang *et al*. 2021), sedimentation (Irawan & Nganro 2016; Noya *et al*. 2016b; Rahmawan *et al*. 2019). The condition of the waters of IAB which is more influenced by anthropogenic nutrient inputs from the mainland through river flow, and the supply of nutrients from the OAB from the influence of upwelling in the Banda Sea which can increase primary productivity and trigger eutrophication in IAB waters (Basit *et al*. 2012; Pello *et al*. 2014, Mahmudi *et al*. 2020); Contamination of heavy metals, Cadmium (Cd) and Cromium (Cr) in sediments, nitrate and phosphate content is already high and in the nutrient contaminated category due to the influence of runoff from land and input from rivers (Ikhsani *et al*. 2016). Human settlements, land use around IAB, and semi-enclosed morphological conditions of IAB, as well as the number of river estuaries are factors that trigger the decline in quality of IAB waters (Gemilang *et al*. 2017; Salamena *et al*. 2021).

High ecological pressure in IAB waters affects the population dynamics of *S. canaliculatus*. Several authors (Manik 1998; Latuconsina *et al*. 2020b) found that the higher fishing mortality and exploitation rate of *S. canaliculatus* without considering the size catch limit resulted in fishing effort are economically unfeasible. Moreover, overexploitation and habitat degradation has caused a decrease in genetic diversity (Toro & Caballero 2005; Markert *et al*. 2010; Martinez *et al*. 2018). Recently, Madduppa *et al*. (2019) reported the occurrence in genetic diversity of the rabbitfish comparing the north and south of Jakarta Bay population in which the environmental influences and the impact of anthropogenic activities showed a greater impact.

This research is a preliminary study using mitochondrial CO1 sequences, to determine whether differences in the physical characteristics of seagrass habitat and geographical distance in IAB can affect the genetic diversity and genetic distance of *S. canaliculatus*. The research results are expected to become scientific information to support habitat-based fisheries management.

## MATERIALS AND METHODS

### Collection Sample

**Figure 1:**
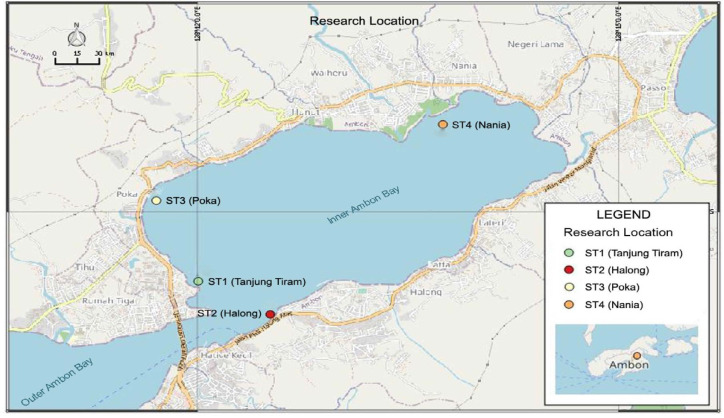
Map of research stations in Inner Ambon Bay Waters (IAB). (Source: Quantum GIS ver. 2.18 ‘las palmas’)

A total of 14 individual *S. canaliculatus* (samples) were collected from fish sampling locations in different seagrass habitats. The first habitat is the seagrass mixed vegetation located at two observation stations; Station 1: Tanjung Tiram with as many as three samples, and Station 2: Halong with as many as four samples. Meanwhile, in the monospecific seagrass meadows, there are two observation stations which are both drained by rivers, namely; Station 3: Poka with as many as three samples, and Station 4: Nania with as many as four samples (Fig. 1).

Mixed vegetation seagrass habitat (five species of seagrass: *Enhalus acoroides, Thalassia hemprichii, Halophila ovalis, Cymodocea rotundata* and *Halodule pinifolia*) at two observation stations. Station 1: Tanjung Tiram (3°39’16.5”S and 128°12’0.43”E) dominated by fine sand substrate, around which mangrove vegetation grows, but not dense because it is very close to residential areas. Station 2: Halong (3°38’32.9”S and 128°12’31.2”E) is dominated by coarse sand substrate, around which there is a coral reef rehabilitation/transplant area. Whereas monospecific seagrass meadows (only one species of seagrass, *Enhalus acoroides*) includes two observation stations which both have river mouths. Station 3: Poka (3°38’36.48”S and 128°11’42.54”E) with a sandy mud substrate, and far from the coastline and has a rehabilitation mangrove area although not extensive. Station 4: Nania (3°37’58.7”S and 128°13’45.1”E) dominated by sandy mud substrate, around which natural mangrove vegetation grows which is quite dense, is located far from the coastline, and has been designated as a conservation area by the government local (Latuconsina *et al*. 2020a).

#### Isolation and purification

Tissue samples from 14 fish were preserved in absolute ethanol. Genomic DNA extraction using the Mini Kit for Tissue Genomic DNA Mini Kit (Gene Aid) Cat. No. GT050 (Vogelstein & Gillespie 1979) according to the Spin-Column protocol with modifications.

#### Amplification and sequencing

The nucleotides of the COI gene were amplified using polymerase chain reaction (PCR). The COI primer used is a universal aquatic biota primer with the primary name COI-F (5′-GGTCAACAAATCATAAAATATTGG-3′) and primary COI-R (5′-TAAACTTCAGGGTGACCAAAAAATCA-3′) with a sequence length of 700 bp (Butet 2003, unpublished). Amplification uses the Labcycler Squence engine.

The volume of the mixed reaction composition is 25 μL with 4.5 μL ddH2O, 12.5 μL My Tag HS Red Mix, 1.5 μL primer forward and 1.5 μL reverse primer and 5 μL template DNA. Temperature PCR is pre denaturation 94°C (3 min), followed by denaturation for 35 cycles at 94°C (45 s), annealing 54°C (1 min), elongation 72°C (30 s), and final extension 72°C (5 min). Further sequencing was carried out in the First Base laboratory (Malaysia), using amplified DNA stored in agarose gel 1.2% (Sambrook *et al*. 1989).

### Data Analysis

Nucleide editing and alignment uses Clustal W in MEGA 10.2.2 program (Kumar *et al*. 2018). Analysis of genetic diversity which includes haplotype diversity (Hd) and nucleotide diversity (π) uses the DnaSP program version 510.01 (Rozas 2009) and Network version 5 (Bandelt *et al*. 1999). Analysis Molecular Variance (AMOVA) and Fixation Index (Fst) were analysed through the Arlequin program (Excoffier *et al*. 1992). Intra and interpopulation genetic distance (D) and phylogenetic tree based on the neighbour-joining (NJ) method with the Kimura 2-parameter (K2P) model with bootstrap 1000 repetition uses MEGA 10.2.2 programme (Kumar *et al*. 2018). Sample identification based on sequence similarity approach was carried out using Barcoding of Life Database (BOLD) (http://www.barcodinglife.org) and GenBank (http://www.ncbi.nlm.nih.gov). The in-group sequences of the COI gene from GenBank consist of *S. canaliculatus* (KJ872545) from China, *S. fuscescens* (EF025185) from China, *S. sutor* (MG677546) from South Korea, and out-group *Lutjanus russellii* (EF514208) from China.

## RESULTS

### Genetic Diversity of White-Spotted Rabbitfish

A total of 14 sequence samples that were BLASTn at NCBI and BOLD Systems showed an average identity of the four populations, representing observation stations from IAB waters (Tanjung Tiram, Halong, Poka and Nania) varied between 98.37% to 98.96% with a query cover of 100% with the species *S. canaliculatus* (KJ872545.1) origin in China.

### Single Nucleotide Polymorphism

Table 1 shows the comparison between COI gene marker sequences results of *S. canaliculatus* found in IAB waters and KJ872545.1 from China. At 700 bp, it was obtained three sites of single nucleotide polymorphisms (SNPs) namely sites 16 (A), 229 (A) and 379 (C).

Based on haplotype and nucleotide variability, the genetic diversity of white-spotted rabbitfish (*S. canaliculatus*) population living in different seagrass habitats in IAB waters is presented in Table 2.

The genetic differentiation of the population between observation stations in IAB waters showed a significant difference between the Fst and AMOVA tests (0.79; *p* < 0.05) (Table 3).

### Haplotype

**Figure 2:**
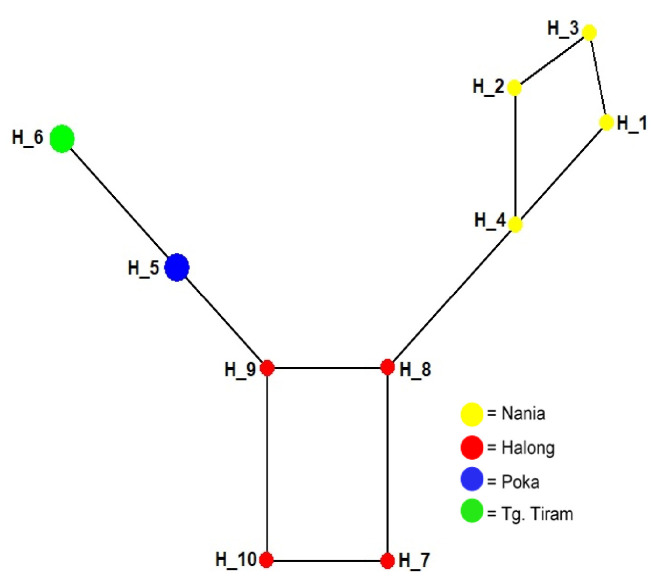
Median joining network of inter-population haplotypes of *S. canaliculatus* in different seagrass habitats in IAB forming three population groups.

The results of the Network Median Joining analysis for the inter-population of *S. canaliculatus* from four seagrass habitats (Fig. 2) show that the haplotype construction forms three clusters.

### Genetic Distance

Intra- and inter-population genetic distances of *S. canaliculatus* in seagrass habitats differed in IAB waters by 0.00–0.37, and 0.40–1.13, respectively. The lowest intra-population genetic distance, 0.00, was found in Tanjung Tiram and Poka, while the highest genetic distance at Halong station was 0.37 (Table 4).

The low inter-population genetic distance, Tanjung Tiram and Poka, were 0.40. The highest was Tanjung Tiram and Nania at 1.13 (see Table 4). Population in Tanjung Tiram and Poka have the closest kinship, it is because the two populations are still in close water areas compared to the distance between the Tanjung Tiram population and the Nania population which is quite far.

### Phylogenetic

**Figure 3:**
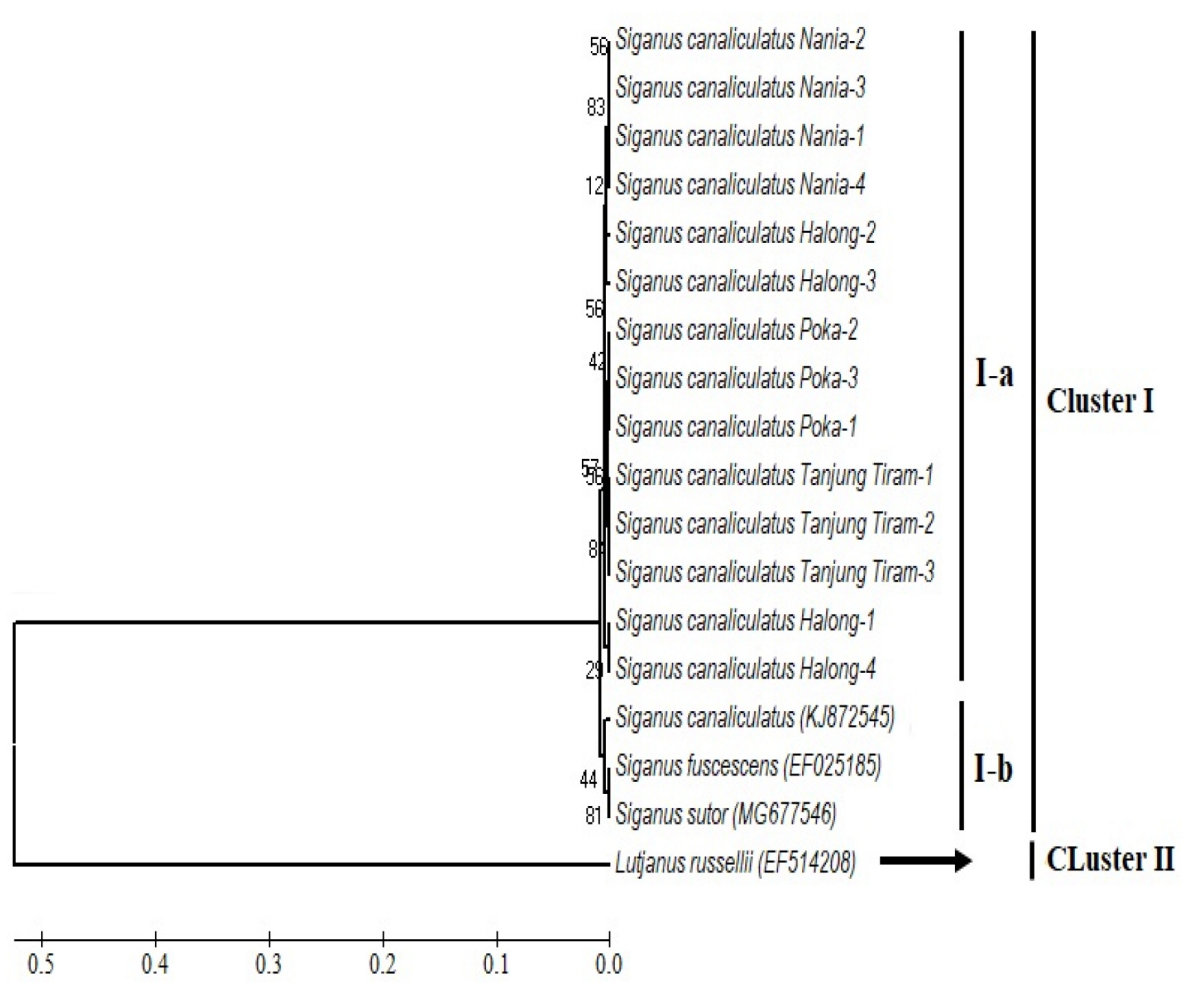
Neighbour-joining (NJ) phylogenetic tree reconstruction using 14 sequences of *S. canaliculatus* based on Kimura 2-parameter model and 1,000 bootstrap replicates.

The interpopulation phylogenetic reconstruction of *S. canaliculatus* from IAB and *Siganus* spp. from other worlds is based on the NJ models with 100 bootstrap repetitions (Fig. 3). It is known that the population of *S. canaliculatus* is far from the population of China and South Korea with bootstrap values of 10 to 85. However, it was seen that two individuals (Halong-1 and Halong-4) were slightly separated from other individuals and clustered differently, with a genetic distance of 0.37 (0.37%) and a bootstrap value of 30.

## DISCUSSION

CO1 gene is a gene that has effectiveness in validating species at the intraspecies and interspecies levels (Ward *et al*. 2005). Species that have a similarity level (> 97%) are the same or closely related species (Hebert *et al*. 2003). The COI gene is a genetic marker that can also provide clear information on changes in the sequence of nucleotide bases between species (Hajibabaei *et al*. 2006; Ward *et al*. 2008). Therefore, it has been widely used for animal identification. In addition, the COI gene can identify reproductively isolated groups.

The various sites are specific locations found exclusively on *S. canaliculatus*, serving as the species barcode within the IAB waters. In addition, specific sites were found at Tanjung Tiram with specific sites [677 (A), 679 (G), 703 (T)], and Nania [693 (G), 714 (A)] can be seen in Table 1, while the Halong and Poka populations do not have specific nucleotide sites. Intra- and inter-population genetic variation in different seagrass habitats is proven by the presence of nucleotide polymorphisms (specific nucleotide sites). This indicates the possibility of gene mutations as the cause of intra- and inter-population variations. Bachry *et al*. (2020) stated that site-specific can be used as the genetic identity of a population. Genetic variation will increase because offspring receive a unique combination of genes and chromosomes from their parents through gene recombination that occurs through sexual reproduction (Indrawan *et al*. 2007; Soewardi 2007; Irmawati 2016).

Halong and Nania populations representing mixed vegetation and monospecific vegetation seagrass habitats each had four haplotypes from four samples with high haplotype (Hd) diversity of 1,000 and nucleotide diversity (π) of 0.004 and 0.002. While in Poka and Tanjung Tiram, each had one haplotype from three samples with low haplotype and nucleotide diversity of 0.000 (Table 2). Nucleotide diversity is the average number of nucleotide differences per site between two randomly selected individuals from a population (Hughes *et al*. 2008). According to Hughes *et al*. (2008) and Mukhopadhyay and Bhattacharjee (2016), the level of genetic diversity within a population can affect survival, productivity, growth, reproduction, population stability, as well as inter-specific interactions within communities, and processes at the ecosystem level.

Inter-population variation is greater than intra-population with values of 78.81% and 21.19%, respectively (Table 3). Madduppa *et al*. (2019) also found inter-population genetic variation (62.04%) which was higher than intra-population (37%) between the Northern and Southern regions of the Seribu Islands, Jakarta, Indonesia, where the diversity between these areas was thought to be influenced by high fishing pressures and anthropogenic activities such as pollution. According to Mukhopadhyay and Bhattacharjee (2016), genetic variation not only affects individual or population fitness, but also contributes to population fitness and successful recovery.

Each observation station that represents a different seagrass habitat certainly has different environmental characteristics. As reported by Latuconsina *et al*. (2020a), that Poka and Nania are characterised by monospecific seagrass habitats, each of which is fed by a river, so that they have similar characteristics in the form of typical turbidity values, dissolved oxygen, chlorophyll-a, and high temperatures. While Tanjung Tiram has high levels of phosphate and nitrate, and together with Halong station has high salinity and pH characteristics, this is possible because there is no river influence which can cause pH and salinity fluctuations in Tanjung Tiram and Halong. Differences in environmental conditions can affect the genetic variation of *S. canaliculatus* populations between observation stations, which in turn has the potential to affect its phenotype. According to Li *et al*. (1993) and Mukhopadhyay and Bhattacharjee (2016), environmental conditions, genetic factors, and genetic interactions with the environment can affect phenotypic differences.

Latuconsina *et al*. (2020a) found higher levels of turbidity in seagrass habitats of monospecific vegetation (Poka and Nania) compared to mixed vegetation (Tanjung Tiram and Halong). This phenomenon is supported by Noya *et al*. (2016b) who estimated that the highest cohesive sediment concentrations occurred in the Poka to Nania areas, where river mouths are the main source of sediment material carrier, with sediment transport rates in IAB waters reaching the range of 1.75 cm–10.01 cm or about 39.9 mm/day. Irawan and Nganro (2016) reported that high sedimentation in IAB waters had threatened the existence of seagrass habitats. This phenomenon is thought to affect the adaptability of *S. canaliculatus* populations to each seagrass habitat with different turbidity and sedimentation pressure, which of course has the potential to affect changes and genetic variation between *S. canaliculatus* populations between seagrass habitats.

The genetic differentiation of intra- and inter-population *S. canaliculatus* populations in seagrass habitats in IAB waters is thought to be influenced by tidal currents that support gene flow through individual migration and distribution of pelagic larvae. The current direction pattern that occurs in IAB waters, according to Fadli *et al*. (2014), is strongly influenced by the tides, where during the tidal phase towards the lowest ebb, the water flows out of the IAB waters, on the other hand at low tide towards the highest tide the water flows into IAB waters. According to Swain *et al*. (2004), genetic differences between groups are an indication of genetic differentiation that is influenced by two factors:

1. Gene flow, namely changes in allele frequencies resulting from the movement of gametes between individuals or groups that can reduce or prevent differentiation.
2. Gene drift refers to random fluctuations in frequency. Alleles arise from the gametes sampling in a finite population and can promote genetic differentiation.

The genetic diversity of the high population of *S. canaliculatus* between seagrass habitats in IAB waters will be very vulnerable if one of the seagrass habitats is damaged or even disappears due to other anthropogenic activity, including the high exploitation of *S. canaliculatus*. According to Martinez *et al*. (2018), life history and habitat characteristics play a role in shaping patterns of genetic diversity in fish, so they should be considered in prioritising species for conservation efforts.

Cluster 1 shows the population of Nania (H1, H2, H3 and H4), Cluster 2 shows the population of Halong (H7, H8, H9 and H10), and Cluster 3 consists of the population of Poka (H5) and Tanjung Tiram (H6). The highest variation of nucleotide sites was found at Halong and Nania stations, each of which represented different seagrass habitats, namely mixed vegetation and monospecific vegetation (Fig. 3). Bramandito *et al*. (2018) reported nine haplotypes from several populations of *S. canaliculatus* from the Seribu Islands, Jakarta, Indonesia. Bachry *et al*. (2019) reported six haplotypes in the aquatic animal population of the abalone species *H. squamata* from the southern waters of Java and Bali. The number of haplotypes and the diversity of haplotypes can affect the genetic diversity of a population (Akbar *et al*. 2014).

Ambon Island has a unique geological history, and consists of two different islands, namely Leihitu in the north, which is a volcanic island, and Leitimur in the south which was formed due to tectonic activity. These two distinct islands are connected by a narrow isthmus (Honthaas *et al*. 1999; Pownall *et al*. 2013). This means that geologically, Inner Ambon Bay (IAB) was once united with Baguala Bay in the eastern part of Ambon Island, which in geological history then separated after the merging of these two islands with the existence of a narrow isthmus. This phenomenon might explain that the Nania station (which is in the position of the narrow isthmus that unites Leihitu and Leitimur) has a specific nucleotide site because it is more isolated with a longer genetic distance compared to the other three observation stations (Tanjung Tiram, Halong and Poka).

Madduppa *et al*. (2019) obtain a close genetic distance (0.00–0.06) in the population of *S. canaliculatus* between stations representing the Southern and Northern parts of the Seribu Islands cluster of Jakarta, Indonesia. Sahabuddin *et al*. (2019) found that the genetic variation of the population of *S. canaliculatus* is strongly influenced by the physical distance of a water area. According to Nei (1987), the value of genetic distance is a value scale that describes the similarity of base sequences in the CO1 gene fragment, the smaller the value, the closer the kinship relationship between the two populations. Irmawati (2016) stated that fish individuals that have close genetic distance or high DNA sequence homologs can be categorised as one population.

The cause of the difference in genetic distance values between populations of *S. canaliculatus* is strongly suspected to be caused by the migration of individual fish supported by different current patterns in the IAB waters. Nurfitri and Putri (2019) simulation to look at current patterns in Ambon Bay shows that surface water masses coming and going from and to the IAB are through the narrow west side (Tanjung Tiram) of the inlet/canal (sill). According to Saputra and Lekalette (2016), the pattern of current directions in IAB waters follows a low tide period at a depth of 5 m which moves from the east to the middle of the bay then southwest towards OAB but most are blocked when crossing the threshold. While the flow of water from the OAB during high tide does not originate from the sill towards the IAB, when it passes through the sill, the current is divided into two different directions: (a) The mass of water entering through the West side (Tanjung Tiram) heading to the northwest (Poka), and at low tide the current pattern from the Poka moves towards the next Poka towards the OAB past the Sill, and (b) on the other water masses to the Southeast (Halong), then moves to the Northeast towards the centre of the IAB.

This phenomenon can answer the absence of specific nucleotide sites between Tanjung Tiram and Poka stations because the current pattern is thought to help distribute pelagic larvae of the *S. canaliculatus* population to connect to each other at the two stations. In contrast, the Poka and Tanjung Tiram stations with the Halong stations are not connected to each other because there is no current that distributes the pelagic larvae of *S. canaliculatus* to be connected between the three stations. This phenomenon can answer the many specific nucleotide sites in Halong station.

Eddy events often occur with different patterns between the tides and ebbs of sea water, as well as between the rainy and dry seasons in IAB waters. As Noya *et al*. (2016) and Noya *et al*. (2019) reported that counterclockwise vertical eddies are dominant in the low tide phase, and in the high tide phase there are clockwise vertical eddies. Water discharge from the estuary that flows into the IAB is thought to be the main factor influencing circulation and eddy patterns in the IAB during the rainy season. Meanwhile, the configuration of topography and tidal parameters during the dry season is thought to be the main factor causing eddies in the IAB. In addition, the dominant anti-clockwise horizontal eddy occurs in the middle of the bay and the eastern part of the IAB, while the dominant clockwise horizontal eddy occurs in the northern part of the IAB. The eddy phenomenon is thought to be one of the physical barriers to the spatial distribution of *S. canaliculatus* pelagic larvae among seagrass habitats, for example between Tanjung Tiram and Poka and Halong, as well as between Nania and other observation stations (Tanjung Tiram, Halong and Poka). There is a relatively large difference in genetic distance between *S. canaliculatus* populations at Nania station and other stations (Table 4), presumably due to the Eddy phenomenon that occurs at several central points of IAB during high and low tide periods as a physical barrier as reported by Noya *et al*. (2016a), and Salamena *et al*. (2021). This phenomenon can also explain the discovery of specific genetic nucleotide sites for *S. canaliculatus* at Halong and Nania stations.

Putra and Pratomo (2019) analysed the direction and speed of currents in the waters of Ambon Bay, at high tide, the current speed tends to be small, namely 0.01 m/s and the current direction only rotates in IAB waters, while at the lowest tide, the current speed range is 0.015 m/s–0.030 m/s with the dominant direction towards OAB waters. According to Fadli *et al*. (2014), movement and current patterns play an important role in changing water masses in Ambon Bay. This phenomenon is thought to play an important role in supporting the distribution of pelagic larvae in different seagrass habitats according to current patterns in IAB waters.

The presence of genetic variation of the *S. canaliculatus* population at each observation station (between seagrass habitats) in IAB waters shows the role of tidal currents as a supporter of the distribution of pelagic larvae in the direction of the current pattern, it also becomes a barrier to the distribution of pelagic larvae that are not in the directions of the current pattern although the distance between adjacent seagrass habitats such as between Tanjung Tiram and Halong stations is only one nautical mile, but separated by a threshold between the OAB, so that the current becomes a barrier to the distribution of larvae and adults of *S. canaliculatus* between adjacent habitats. Rabbitfish (*Siganus* spp.), according to Fisher *et al*. (2005), showed wide dispersal potential supported by ocean currents, with strong swimming ability at speeds of 34.2 cm/s–87.1 cm/s (mean 67.1 ± 8.9) during the pelagic larval stage (29.5 mm TL). This phenomenon will certainly support spatial distribution. *S. canaliculatus* in IAB waters are supported by tidal currents. As (Hsu & Gwo 2017) found ocean currents that have a positive contribution in efforts to recover the population of *S. fuscescens* from extreme temperature stress.

Grant and Bowen (1998), stated that marine fish generally show low levels of genetic differentiation between geographic areas due to the potential for higher dispersal during the pelagic egg and larval stages or during the adult phase coupled with the absence of physical barriers to movement between adjacent sites. According to (Lin & Liu 2008), the close genetic distance is also caused by currents, the high dispersal ability of biota larvae, and the availability of supportive habitats (Huyghe & Kochzius 2018). Madduppa *et al*. (2019) revealed that genetic distance is an assessment of kinship relationships between populations from several locations, where the lower the value of genetic distance obtained indicates a very close relationship between locations, and the higher the value indicates a fairly distant relationship between locations.

Based on the phylogenetic tree, the *Siganus* spp. and Outgrup *Lutjanus russellii* depicts two main clusters. Cluster 1 shows two main subclusters consisting of subcluster (1-a) of intrapopulation *S. canaliculatus* from IAB and subcluster (1-b) from genbank (*S. canaliculatus, S. fuscescens, S. sutor*) originating from other geographical areas (Fig. 3).

Interestingly, the intrapopulation at Halong is phylogenetically separated among the four sequences of *S. canaliculatus* (Fig. 3), where Halong-2 and Halong-3 are more closely related to the Nania population, while Halong-1 and Halong-4 are more closely related to the Tanjung Tiram population. It is suspected that the geographical condition of Halong station is more open and faces an inlet that separates IAB waters from OAB, so that the opportunity for gene migration from inside and outside the IAB waters is greater through crossbreeding. According to Soewardi (2007), genetic differences between populations are supported by gene flow between sub-populations and genetic drift from certain sub-populations.

The accuracy of the kinship test based on the CO1 gene sequence can be relied on. Tree clades represent relationships between units by tracing the paths of descent from ancestors. Li and Graur (2000) revealed that the branch length indicates the number of evolutionary changes that occurred between the two clusters. The diversity of CO1 gene sequences has the potential to identify species and DNA barcodes (Hebert *et al*. 2003; Hebert & Gregory 2005).

The results of this study confirm that the *S. canaliculatus* intraspecies is phylogenetically separate from other subspecies of *S. canaliculatus*, although the results are still in the same cluster. Meanwhile, *S. canaliculatus* intraspecies is still a sub-species of the genus *Siganus*. Our suggestion is to collect more molecular data from this species distribution to establish a stronger status for protected taxa.

## CONCLUSION

The pattern and direction of tidal currents with the eddy phenomenon as a link or barrier to spatial distribution and connectivity of *S. canaliculatus* larvae between seagrass habitats, as well as the presence of different anthropogenic pressures in each seagrass habitat, are thought to influence the genetic characteristics (genetic diversity, genetic variation, genetic differentiation and genetic distance) of *S. canaliculatus* populations in IAB waters.

Preliminary research using mitochondrial CO1 sequences can at least explain that the characteristics of seagrass habitat and its geographical location in narrow bay areas such as in IAB can affect the diversity, variation, differentiation and genetic distance of *S. canaliculatus* populations. Thus, further in-depth and comprehensive research is still needed regarding population genetics and inter-population connectivity of *S. canaliculatus* in the waters of IAB and outside IAB, with a more representative number of samples using microsatellites, so that it is expected to complement the results of the preliminary research that has been done.

The results of this research can provide information about the urgency of habitat-based fisheries management of *S. canaliculatus* through the development of conservation areas based on the characteristics of seagrass habitats and the biological characteristics of *S. canaliculatus* populations, including their genetic characteristics, to support sustainable utilisation in IAB waters.

## Figures and Tables

**Table 1:** Single nucleotide polymorphism of the *S. canaliculatus* from IAB and China.

Species/Access code	Nucleotide site															

			2	3	5	6	6	6	6	6	6	6	7	7	7	7
	1	2	2	79	0	1	1	4	7	7	8	9	0	0	1	1
	6	0	9	9	5	4	9	1	7	9	4	3	1	3	3	4
*S. canaliculatus* (KJ872545.1)*	G	C	G	T	C	T	A	A	G	T	G	A	T	C	G	G
*S. canaliculatus* Tanjung Tiram-1	A	T	A	C	T	C	.	.	**A**	**G**	A	.	C	**T**	.	.
*S. canaliculatus* Tanjung Tiram-2	A	T	A	C	T	C	.	.	**A**	**G**	A	.	C	**T**	.	.
*S. canaliculatus* Tanjung Tiram-3	A	T	A	C	T	C	.	.	**A**	**G**	A	.	C	**T**	.	.
*S. canaliculatus* Halong-1	A	.	A	C	.	.	.	.	.	.	.	.	.	.	A	.
*S. canaliculatus* Halong-2	A	.	A	C	T	C	.	.	.	.	A	.	.	.	A	.
*S. canaliculatus* Halong-3	A	.	A	C	T	C	.	.	.	.	A	.	.	.	.	.
*S. canaliculatus* Halong-4	A	.	A	C	.	.	.	.	.	.	.	.	.	.	.	.
*S. canaliculatus* Poka-1	A	T	A	C	T	C	.	.	.	.	A	.	C	.	.	.
S*. canaliculatus* Poka-2	A	T	A	C	T	C	.	.	.	.	A	.	C	.	.	.
*S. canaliculatus* Poka-3	A	T	A	C	T	C	.	.	.	.	A	.	C	.	.	.
*S. canaliculatus* Nania-1	A	.	A	C	T	C	.	G	.	.	A	**G**	C	.	A	**A**
*S. canaliculatus* Nania-2	A	.	A	C	T	C	G	.	.	.	A	**G**	C	.	A	**A**
*S. canaliculatus* Nania-3	A	.	A	C	T	C	G	G	.	.	A	**G**	C	.	A	**A**
*S. canaliculatus* Nania-4	A	.	A	C	T	C	.	.	.	.	A	**G**	C	.	A	A

*Note:*

* = GenBank origin species.

**Table 2:** Genetic diversity of *S. canaliculatus* on seagrass habitats in IAB..

Type of seagrass habitat	Research station	*n*	Genetic diversity		

			Hn	Hd	π
Mixed vegetation	Tanjung Tiram	3	1	0.000	0.000
	Halong	4	4	1.000	0.004
Single vegetation	Poka	3	1	0.000	0.000
	Nania	4	4	1.000	0.002

*Note: n* = number of samples, Hn = number of haplotypes, Hd = Haplotype diversity, π = nucleotide diversity

**Table 3:** Genetic differentiation (Fst) of inter and intra-population on *S. canaliculatus* based on different seagrass habitats in IAB.

Source of variation	d.f.	Variation (%)	Fst	*p*-value
Inter-population	3	78.81	0.79	<0.05
Intrapopulation	10	21.19		
Total	13			

*Note:* d.f = degree of freedom. Fst = Genetic differentiation

**Table 4:** Genetic distances of *S. canaliculatus* CO1 gene based on Kimura 2-parameter model.

Site	Tanjung Tiram	Halong	Poka	Nania
Tanjung Tiram	0.00*			
Halong	1.00	0.37*		
Poka	0.40	0.58	0.00*	
Nania	1.13	0.86	0.73	0.17*

*Note:*

* = intra-population mean genetic distance of *S. canaliculatus* in IAB.
